# Models and Casts

**Published:** 1857-04

**Authors:** H. R. Smith


### ARTICLE XV.

### *Models and Casts.* By H. R. SMITH.

IN my last article, I endeavored to guide the student over the forms of taking impressions of the mouth in wax and plaster, and which, if he rightly understands, he is now ready to proceed with me as I pass on to describe the next step of our operation.

If our impression has been taken in wax, we with a camel's-hair brush give it a delicate coat of sweet oil. If in plaster, we first give it a coat of varnish, and then one of oil, to keep the plaster from adhering to the impression.

We then surround it with a band of paper, waxed cloth or sheet lead, the width that we wish our model to be thick.

After this is done, we prepare our plaster by mixing it with water to the consistence of pretty thick cream. We first place a small quantity in the impression, and see that it fills all of the small indentations and impressions made by remaining teeth, which is done by blowing on the plaster and by jarring the bench near our impression.

Unless that is done, small air holes will be found in our model, which will make it imperfect.

After that is done, pour in the remaining plaster, to make it as deep as we wish.

The impression is then allowed to rest until the plaster has become well hardened, which time will vary according to the quality of our plaster.

After the plaster is sufficiently hardened, it should be removed very carefully from the impression, and trimmed and varnished.

For this purpose, either the white or yellow varnish may be used. The white is made by dissolving gum sandarach, and the yellow, gum shellac in alcohol. It should be applied with a camel's-hair brush, and spread lightly and evenly over the model.

When the varnish is dry, then the model will be ready for use. If, however, a chamber is to be used in the plate, it is best to prepare for it at this stage of the operation.

As almost every dentist has his favorite way of preparing his air chamber, it would be useless to attempt to give all of them, but I will give two ways of performing the operation.

The first is when the chamber is stamped into the plate—the second, where the chamber is formed by the shape being cut out of the first or main plate, and afterwards forming over it a second or false plate, at a little distance from the first, giving the lingual surface the appearance of a plate without any chamber.

When we wish to stamp a chamber into the plate, we take a piece of lead rolled out to the thickness we wish our chamber, then make it the shape we wish, and with a little wax or gutta percha, fasten it on to the plaster model in the situation where we wish our chamber to be in our plate.

After this is done, spread a light coat of varnish over the lead and surface of the model.

The next step in our operation is to prepare our sand for use in moulding.

The best sand for our use is that which has been used for some time in a foundry, and become fine. The sand should first be passed through a fine sieve, to remove all coarse particles and sticks. After the sand is sifted, then water should be sprinkled on it from a sprinkling pot with very fine holes, care being taken that too much be not put on, lest it become too wet. Then rub and work the sand until it is thoroughly tempered. It is well to let it stand a short time before it is used, for it works better.

This is always done by those who cast fine work in iron and brass, as the mould is much smoother and perfect than it would be if used when first prepared. And as a perfect cast is of the greatest importance to the dentist, it is better to use all precautions in our power.

Now place the model on its base on the moulding table, and place the moulding box around it. The sand should be rubbed fine or sifted as it is placed over the model, otherwise the metal when poured in will sink deeper into the sand in some points than at others, which will of course, make the metallic model or cast imperfect. Great care should be taken in pressing the sand around the model.

If the sand is left too loose, the metal will sink into it, and the cast will be rough, while, if the sand is pressed too hard, the steam caused in the wet sand will not be able to escape through the sand, but will pass up through the metal, causing ebullition, thereby making the cast full of holes, and imperfect. As no rule can be laid down for this part of the operation, practice only can teach how it is done.

After the sand is compressed, the box should be turned over, and the model rapped gently with a small mallet or hammer, to loosen it in the sand. After this is done, the model can be easily withdrawn. Care should be taken not to rock the model while drawing, else the plate, when stamped, will rock in the mouth.

A little pulverized charcoal in a muslin bag may now be dusted over the mould, and any excess blown off with the mouth. This will absorb the moisture on the surface, and give a smooth cast.

The mould should now be covered up, to prevent any damage, while we prepare our metal for pouring.

At this stage of the operation, we must choose what metal we will use for our casts.

Let us first consider what qualities we want combined, to give us the most perfect cast. In the first place, we want that metal, or combination of metals, that will have the least shrinkage, for if our metal shrinks very much, as a matter of course, our plate when stamped will be too small for the mouth.

Secondly, we want hardness in our male cast, otherwise, when we stamp the plate, the metal will yield, and be so bruised and battered down, that the rugæ of the mouth will not be represented on the plate.

And thirdly, we want a metal that will, combining these qualities, give us the smoothest cast.

Nearly every dentist has his favorite metal for his casts, and each, in turn, are tried, and laid aside by others. One prefers brass, another cast iron, another zinc, and others a combination of several metals. As for iron and brass, the difficulty of working them in general practice would exclude their use, even if in many respects they were considered preferable.

Type metal makes a very perfect and sharp cast, but it is too soft to resist the plate while stamping. Block tin makes a sharp cast, but like type metal, it is too soft to answer our purpose. Zinc is good on account of shrinkage and hardness, but owing to the large size of its crystals, makes a rough cast. Antimony has hardness and small shrinkage, but too brittle to bear the necessary force to stamp the plate.

I will now give my experience in regard to a combination of these metals to serve our purpose.

My first preference is for block tin six parts, metallic antimony one part. This will give a sharp cast, and at the same time one quite hard.

Great care must be taken when first melting these together to prevent oxydation, for the antimony requires so great a heat to melt, that if care is not used, much of it may oxydize away. During the melting, wax or tallow should be occasionally thrown in, to act as a deoxydating agent, and also as a flux to assist the antimony and tin to melt and combine.

My second choice is, zinc five parts, block tin one part. The zinc gives hardness, and the tin smoothness, and a good cast is the result.

And my third choice would be clear zinc, which, being alone, is not liable to the objection of any of the combinations, that of having the proportions destroyed by oxydation.

For the counter or female cast, a yielding metal is necessary, and for this purpose we prefer, first, lead; secondly, type metal.

To ensure a perfect fit, I use two sets of casts, one to first bend and partially stamp, the second to finish stamping. The first becomes very much bruised and defaced, but the second brings out in bold relief all of the rugæ and inequalities of the mouth.

The casts should be made very heavy, to prevent springing and getting out of place.

Before turning the metal into the mould, it should, after removal from the fire, be stirred with a piece of wood, until crystallization commences, then pour gently into the heel of the mould.

After the cast is sufficiently cooled and removed from the sand and cleaned, it should be well coated with Spanish whiting and water, and well dried. This will prevent the other metal, when poured, from adhering. After surrounding our male cast with our band or moulding box, we then pour our lead, which has been previously partially cooled, as previously directed for the male cast. Then, when cooled, our casts or dies are ready for use.—*Dent. Register.*

